# Chiral Separation of Stilbene Dimers Generated by Biotransformation for Absolute Configuration Determination and Antibacterial Evaluation

**DOI:** 10.3389/fchem.2022.912396

**Published:** 2022-05-31

**Authors:** Robin Huber, Laurence Marcourt, Luis-Manuel Quiros-Guerrero, Alexandre Luscher, Sylvain Schnee, Emilie Michellod, Verena Ducret, Thilo Kohler, Karl Perron, Jean-Luc Wolfender, Katia Gindro, Emerson Ferreira Queiroz

**Affiliations:** ^1^ School of Pharmaceutical Sciences, University of Geneva, CMU, Geneva, Switzerland; ^2^ Institute of Pharmaceutical Sciences of Western Switzerland, University of Geneva, CMU, Geneva, Switzerland; ^3^ Departement of Microbiology and Molecular Medicine, University of Geneva, Geneva, Switzerland; ^4^ Mycology Group, Plant Protection Research Division, Agroscope, Nyon, Switzerland; ^5^ Microbiological Analysis Platform, Microbiology Unit, Department of Botany and Plant Biology, University of Geneva, Geneva, Switzerland

**Keywords:** biotransformation, *Botrytis cinerea Pers.*, stilbene dimers, enantiomers, chiral-HPLC, ECD calculations, *Staphylococcus aureus*, MRSA

## Abstract

A series of complex stilbene dimers have been generated through biotransformation of resveratrol, pterostilbene, and the mixture of both using the enzymatic secretome of *Botrytis cinerea* Pers. The process starts with achiral molecules and results in the generation of complex molecules with multiple chiral carbons. So far, we have been studying these compounds in the form of enantiomeric mixtures. In the present study, we isolated the enantiomers to determine their absolute configuration and assess if the stereochemistry could impact their biological properties. Eight compounds were selected for this study, corresponding to the main scaffolds generated (pallidol, leachianol, restrytisol and acyclic dimers) and the most active compounds (*trans*-δ-viniferin derivatives) against a methicillin-resistant strain of *Staphylococcus aureus* (MRSA). To isolate these enantiomers and determine their absolute configuration, a chiral HPLC-PDA analysis was performed. The analysis was achieved on a high-performance liquid chromatography system equipped with a chiral column. For each compound, the corresponding enantiomeric pair was obtained with high purity. The absolute configuration of each enantiomer was determined by comparison of experimental and calculated electronic circular dichroism (ECD)*.* The antibacterial activities of the four *trans*-δ-viniferin derivatives against two *S. aureus* strains were evaluated.

## Introduction

Resveratrol dimers are a major class of complex stilbenoids mainly distributed in five plant families, namely, Vitaceae, Leguminosae, Gnetaceae, Dipterocarpaceae, and Cyperaceae (Sotheeswaran and Pasupathy, 1993; Shen et al., 2017). These compounds are commonly biosynthesized by regioselective oxidative coupling of two units of stilbene monomers (Keylor et al., 2015; Shen et al., 2017). Stilbene dimers were found to display wide biological properties, such as anticancer, antifungal, antibacterial, anti-HIV, and antioxidant activities (Xue et al., 2014; Shen et al., 2017). Despite the interest in these biological activities, stilbene dimers are difficult to obtain due to their low concentration in plants and their structural complexity, which makes their synthesis challenging (Keylor et al., 2015).

Recently, we have developed an original biotechnological process based on the use of an enriched fraction of enzymes from fungi (“fungal secretome”) for the transformation of conventional stilbenes into complex stilbene dimers with interesting biological activities (Gindro et al., 2017; Righi et al., 2020). The generation of these compounds is supposed to proceed through phenoxy radical couplings probably generated by laccases present in the enzymatic secretome of *Botrytis cinerea* Pers. (Langcake and Pryce, 1977; Keylor et al., 2015). Although this process involves enzymes, the compounds obtained are enantiomeric mixtures. Indeed, laccases generate planar phenoxy radicals which then combine randomly and without enantioselectivity (Langcake and Pryce, 1977; Keylor et al., 2015).

Some of the compounds obtained presented remarkable biological activities against methicillin and vancomycin resistant strains of *Staphylococcus aureus* (Righi et al., 2020). These compounds were originally tested as mixtures of enantiomers. To determine if the activity is due to only one of the enantiomers, a chromatographic separation using a chiral column must be employed.

In this context, the present article shows the investigation using chiral chromatography of a series of stilbene dimers obtained by biotransformation using the enzymatic secretome of *B. cinerea* Pers. The enantiomeric pair of each compound was separated, collected, and submitted to spectroscopic analysis. Their absolute configuration was determined by electronic circular dichroism (ECD)*.* Finally, the biological activities of the main active compounds (*trans*-δ-viniferin scaffold) and their pure enantiomers were evaluated against methicillin-susceptible and -resistant *S. aureus* strains.

## Results and Discussion

In our previous work, a series of biotransformation reactions were performed on resveratrol, pterostilbene, and a mixture of both by using the secretome of *B. cinerea* Pers. (Gindro et al., 2017; Righi et al., 2020). The generated compounds from these reactions were purified by a high resolution reverse-phase chromatographic approach combining isolation using semi-preparative HPLC, dry load injection to maintain resolution (Queiroz et al., 2019), and multiple on-line detection of the collected fractions (UHPLC-PDA-ELSD-MS) (Righi et al., 2020). Using this approach, most of the compounds were obtained in one-step with a high degree of purity. This chemoenzymatic approach generated an important number of derivatives classified in five main scaffolds named *trans*-δ-viniferin, pallidol, leachianol, restrytisol and acyclic dimer.

**FIGURE 1:**
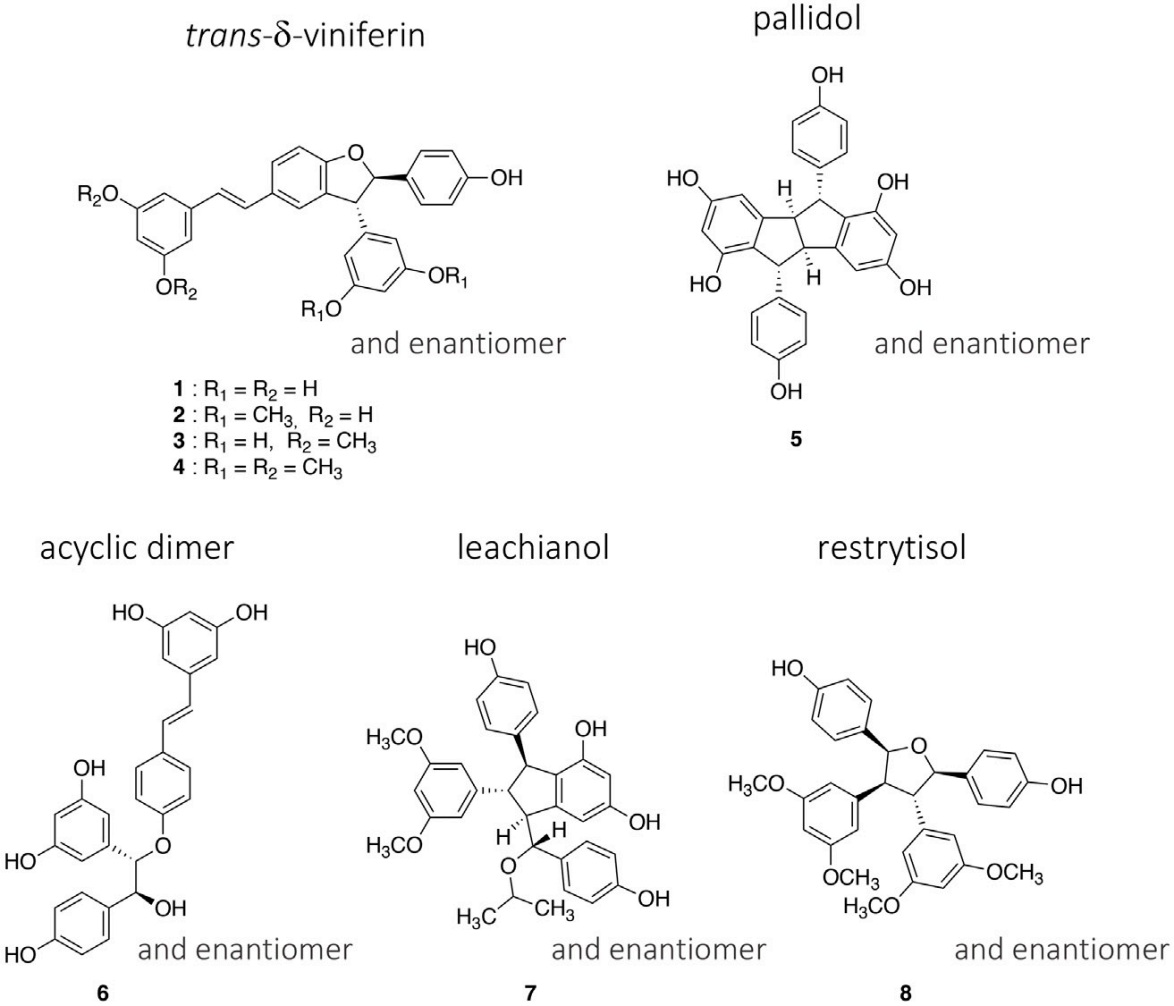
Structures of the four *trans*-δ-viniferin derivatives (**1**–**4**) and the four other selected compounds (**5**–**8**) representing the main scaffolds obtained by the biotransformation of resveratrol and pterostilbene with the enzymatic secretome of *Botrytis cinerea* Pers.

In this work, in addition to the four *trans*-δ-viniferins which had high antibacterial activities, one representative of each type of scaffold was selected (Figure 1). The purity of these compounds (**1**–**8**) obtained in the previous work was checked by UHPLC-PDA-ELSD-MS and by NMR (data not shown). The relative stereochemistry of each scaffold was already described (Huber et al., 2022) and can be summarized as follows: a *trans* configuration of H-7′/H-8′ protons for the *trans*-δ-viniferin (**1**–**4**), a *trans*-*cis*-*trans* configuration of H-7/H-8/H-8′/H-7′ protons for the pallidol (**5**), and a *trans*-*trans*-*cis* configuration of H-7/H-8/H-8′/H-7′ protons for the restrytisolB (**8**). The acyclic dimer scaffold exists in *threo* or *erytho* form, the selected compound being *threo* (**6**) while for the leachianol (**7**) the H-7′/H-8′/H-8/H-7 protons are in *trans*-*trans*-*trans* configuration, but the configuration of C-7 could be different allowing to define the F series (with the phenol on the side of H-10′/H-14′) and the G series (with the phenol on the side of H-14). The selected compound (**7**) belongs to the G series.

For the isolation of each enantiomeric pair, the first step was to choose a suitable HPLC chiral column. A series of columns were screened (see experimental section) and the best results were obtained with a Chiralpak^®^ IB N-5 column. This column is packed with cellulose substituted with *tris* (3,5-dimethylphenylcarbamate) immobilized on a 5 μm silica gel. Separations were performed in isocratic mode using mixtures of heptane and EtOH containing 0.1% diethylamine, with a PDA detection at 235, 280, and 310 nm. All steps, from optimization to compounds isolation, were performed on an analytical column due to the high cost of preparative ones.

### Enantiomeric Separation of the *Trans*-δ-Viniferin Derivatives for Biological Assays

The work was first done on the four compounds belonging to the *trans*-δ-viniferin scaffold. For these compounds, the goal was to obtain at least 1 mg of each pure enantiomer to be able to run biological assays and determine if the anti-MRSA activity of each individual enantiomers will significantly differ from the one reported for the enantiomeric mixture. This is of particular interest for compound **3** which showed an anti-MRSA activity almost as high as vancomycin (Righi et al., 2020).

The isolation of the different enantiomers was performed using an analytical HPLC Chiralpak^®^ IB N-5 column (250 × 4.6 mm i. d., 5 µm; Daicel). Separation was performed in isocratic mode using a mixture of heptane and EtOH containing 0.1% diethylamine as mobile phase, and compounds were manually collected after the PDA detector. For each compound, various isocratic runs were tested to select the most efficient one. After optimization, concentrated solutions were prepared (ca. 5–15 mg/ml in dichloromethane/ethyl acetate mixtures, depending on the solubility of each compound). As the isolation was performed on an analytical column, a series of injections with increasing injected amounts of sample were performed to determine the maximum loading capacity for each pair of enantiomers. Due to the unusually high volumes of solvent injected for an analytical column, the peak shape was strongly distorted. Based on two parameters (solubility and peak resolution), the maximal injectable amount was defined for each compound. It was for example possible to inject 1.25 mg (100 μl, 12.5 mg/ml) of compound **3** and separate the two enantiomers without overlapping of the peaks. In the case of compound **1**, the loading capacity of the column was lower, allowing an injection of a maximum of 0.4 mg (40 μl, 10 mg/ml). In this case, more injections were necessary to obtain enough material for the chemical and biological analysis.

**FIGURE 2:**
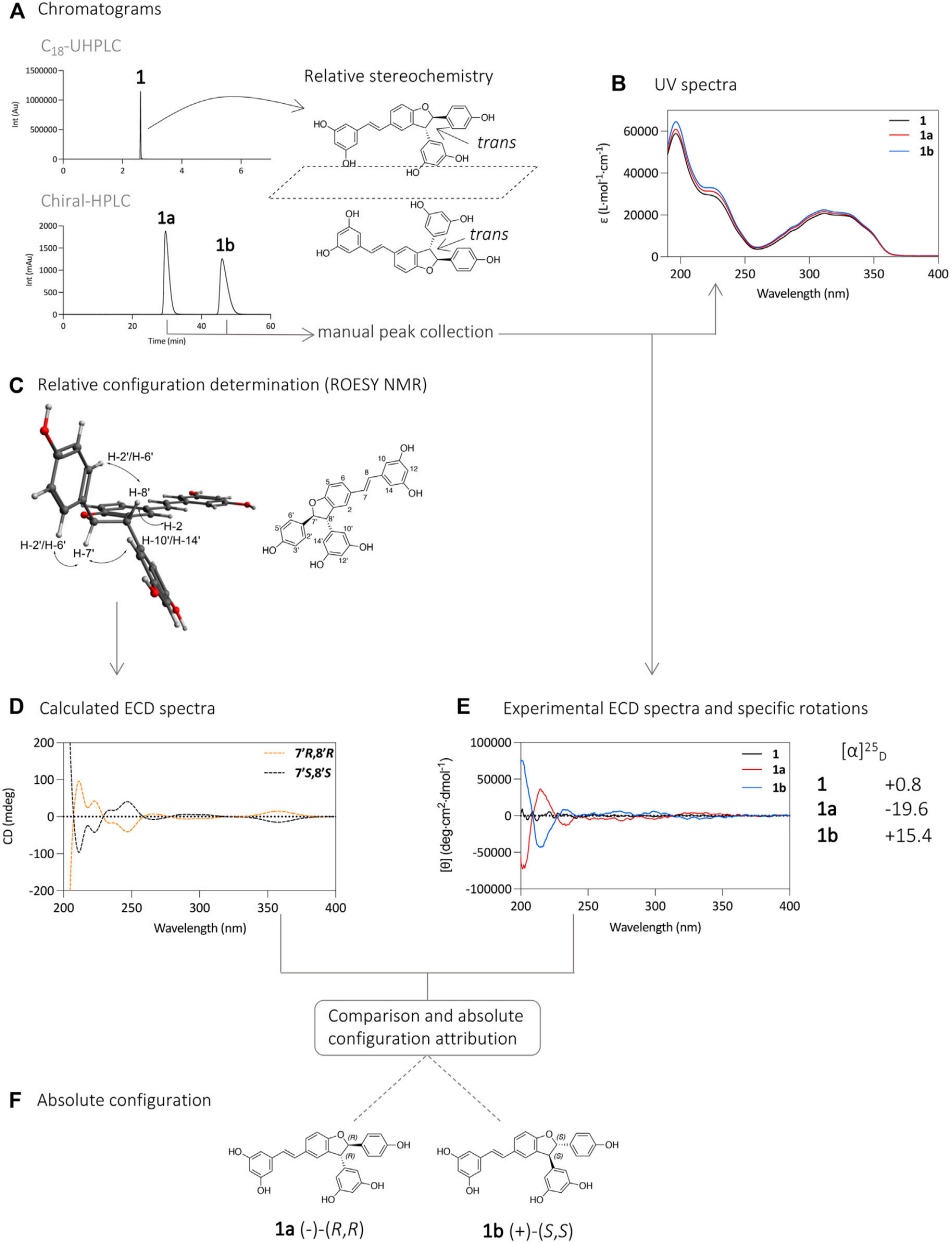
**(A)** UHPLC-PDA analysis of **1** using an Acquity UPLC BEH C_18_ column (50 × 2.1 mm i. d., 1.7 µm; Waters), and separation of its enantiomers **1a** and **1b** on HPLC-PDA using an Chiralpak^®^ IB N-5 column (250 × 4.6 mm i. d., 5 µm; Daicel). **(B)** UV spectra of compounds **1, 1a**, and **1b**. **(C)** Relative configuration determination by ROESY NMR experiment. The spectrum with the correlations is presented in Supplementary Figure S1. **(D)** Calculated ECD spectra. **(E)** ECD and specific rotation ([α]_D_) analysis of compounds **1, 1a**, and **1b**. **(F)** Determination of the absolute configuration of compounds **1a** and **1b**.

Using this approach, the enantiomers of compounds **1**–**4** were successfully separated and obtained in sufficient amount for chemical and biological evaluation. The four enantiomeric pairs obtained were monitored by UHPLC-PDA-ELSD-MS, NMR, and chiral-HPLC-PDA to ensure their purity. Their optical properties were studied by optical rotation and electronic circular dichroism (ECD). The latter were compared to calculated ones and to the literature to determine their absolute configurations (Han et al., 2014; Gavezzotti et al., 2015). The workflow is detailed below for compound **1** as an example (Figure 2).

Analysis of compound **1** by UHPLC-PDA showed a single chromatographic peak corresponding to the compound previously isolated as a racemic mixture. Analysis using a chiral HPLC column confirmed this by the presence of two peaks of similar intensities suggesting that the two enantiomers are present in similar proportions (Figure 2A). The UV spectrum of each chromatographic peak was measured and found to be the same as expected (Figure 2B). The purified enantiomers **1a** and **1b** were subjected to specific rotation ([α]_D_) and ECD analysis to determine the absolute configuration of the 7′ and 8′ carbons (Figure 2E). The specific rotation of compound **1a** was −19.6, and that of compound **1b** was 15.4, while compound **1** had a near zero value of 0.8. These observed values suggest an effective chiral separation of the two optically pure enantiomers. These compounds were then subjected to ECD analysis, and the obtained spectra showed opposite curves with identical magnitude confirming the separation of the two enantiomers, compared to the flat spectrum obtained from compound **1** (Figure 2E).

The experimental ECD spectra were compared to calculated ones, based on the relative configuration determined by NMR. Briefly, NMR analysis of the ROESY spectrum of **1** showed correlations from H-7′ to H-10′/H-14′ and from H-8′ to H-2′/H-6′ indicating a *trans* relative stereochemistry between H-7′/H-8′ (Figure 2C, Supplementary Figure S1). This indicates that the two carbons have the same absolute stereochemistry (*R,R* or *S,S*).

To determine the absolute configuration of the carbons 7′ and 8′ for **1a** and **1b**, the structures with the relative configuration proposed by the NMR were modelled and energetically optimized *in vacuo* using B3LYP hybrid function with a 6–31G (d,p) basis (Stephens et al., 1994; Nugroho and Morita, 2013; Mándi and Kurtán, 2019). All possible conformers were calculated for each molecule and subsequently optimized using the same function and basis set as before but in a SCRF (self-consistent reaction field) model in MeCN. After a cut-off of 4 kcal/mol in energy, the selected conformers were modelled using a TD-DFT (time dependent density functional theory) method with a hybrid function B3LYP and an extended basis def2svp, under a SCRF model in MeCN (Nugroho and Morita, 2013). After this step, the ECD spectra of the two enantiomers were simulated and compared with those of the compounds experimentally obtained. The calculated ECD for the 7′*R*, 8′*R* stereoisomer showed an excellent fit with the experimental data of **1a**, with positive CE (Cotton Effect) around 215 and 320 nm and negative CE around 250 and 300 nm. The other stereoisomer, 7′*S*, 8′*S*, displayed a mirror image. The positive CE around 215 nm in the experimental spectrum was likely due to a π → π* transition in the phenyl moieties, while the CE around 300 nm were likely due to the extended π conjugated system. From this comparison it was possible to assign the (-)-(*R,R*) configuration to compound **1a** (*t*_R_ 30 min) and (+)-(*S,S*) to compound **1b** (*t*_R_ 46 min) (Figures 2D–F). These attributions are consistent with those found in the literature based on ECD spectra of ε-viniferin and 1,2-diaryldihydrobenzofuran (Lins et al., 1986; Kurihara et al., 1990; Bhusainahalli et al., 2012; Han et al., 2014; Gavezzotti et al., 2015).

**FIGURE 3:**
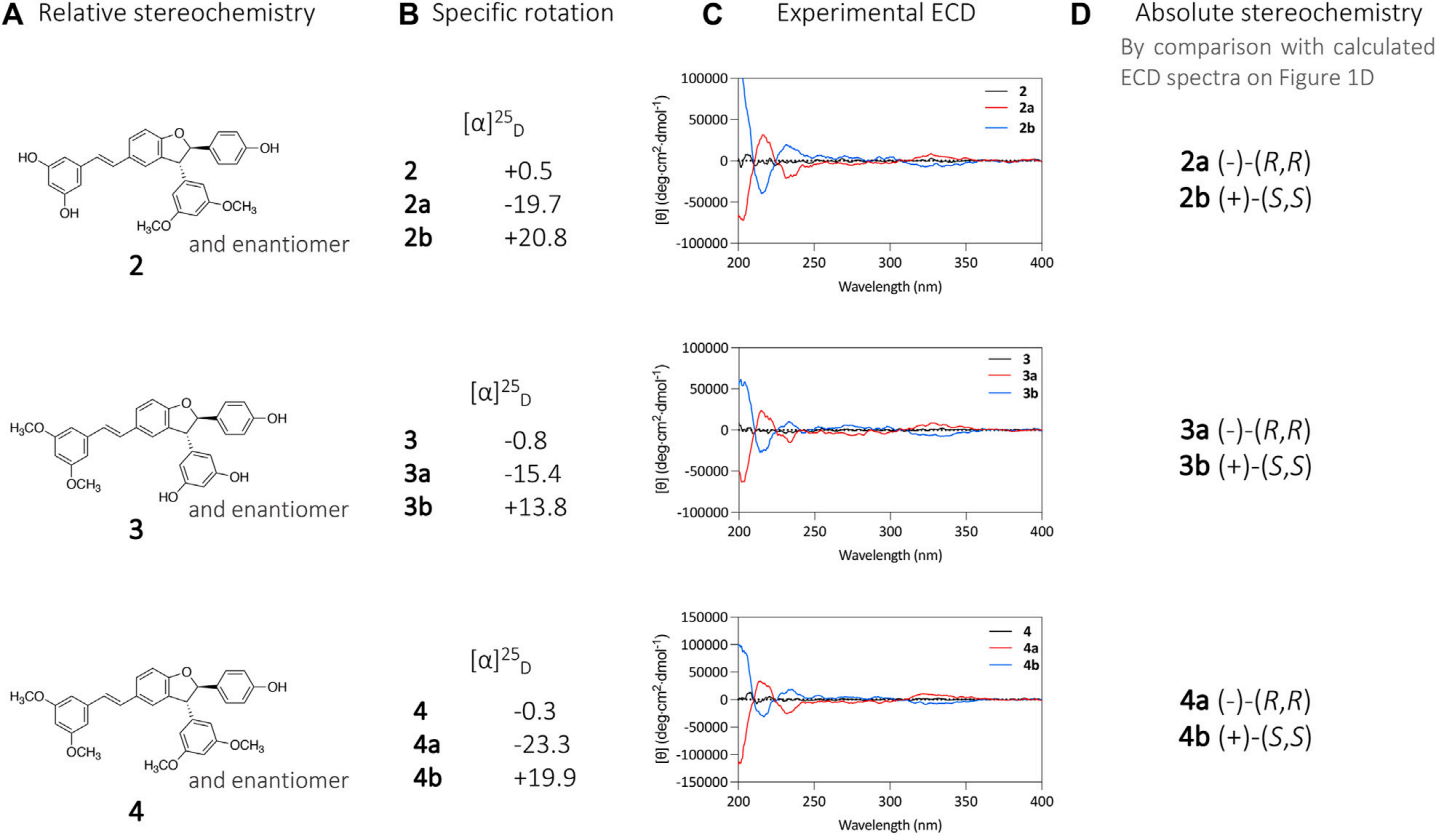
**(A)** Relative stereochemistry of compounds **2**, **3**, and **4** defined by the NMR analysis. **(B)** specific rotation ([α]_D_) and **(C)** and ECD analysis of compounds **2a**, **2b**, **3a**, **3b**, **4a**, and **4b**. **(D)** Absolute stereochemistry attribution based on calculated ECD spectra.

The same procedure described above for compound **1** was applied for the chiral isolation and the absolute configuration determination of the enantiomeric pairs of compounds **2**, **3**, and **4**. This allowed the characterization of compounds **2a** (−)-(*R,R*), **2b** (+)-(*S,S*), **3a** (−)-(*R,R*), **3b** (+)-(*S,S*), **4a** (−)-(*R,R*) and **4b** (+)-(*S,S*) (Figure 3). A summary for each compound with the chromatographic and spectroscopic details can be found on Supplementary Figure S2 (compound **1**), Supplementary Figure S3 (compound **2**), Supplementary Figure S4 (compound **3**) and Supplementary Figure S5 (compound **4**).

It is well known that enantiomers of a chiral drug can exhibit different pharmacological behavior in a chiral environment such as a receptor (McConathy and Owens, 2003). Since some of the *trans*-δ-viniferins investigated in this study presented remarkable antimicrobial activities against resistant strains of *S. aureus* (Righi et al., 2020), we decided to investigate the activities of their respective enantiomers.

For this purpose, the minimal inhibitory concentrations (MICs) of compounds **1**, **2**, **3**, and **4** and their respective purified enantiomers (**1a**, **1b**, **2a**, **2b**, **3a**, **3b**, **4a**, and **4b**) were determined against a methicillin-susceptible and a methicillin-resistant strain of *S. aureus* (Table 1). The *trans*-δ-viniferin derivatives **2** and **3** showed comparable potent activities against both *S. aureus* strains, in agreement with our previous results (Righi et al., 2020). The enantiomers **2a**/**2b** and **3a**/**3b** showed similar activities as their respective racemic mixtures. This demonstrate that the enantiomeric form of these *trans*-δ-viniferins has no influence on the bioactivity and that the possible targets do not require a specific chiral form.

**TABLE 1:** Minimum Inhibitory Concentration (MIC) of compounds **1** to **4** against a methicillin susceptible (MSSA) and a methicillin resistant (MRSA) strain of *Staphylococcus aureus*.

Compounds	*S. aureus* MSSA newman (µM)	*S. aureus* MRSA COL (µM)
**1** (rac^a^)	16	16
**1a** (−)-(*R,R*)	32	32
**1b** (+)-(*S,S*)	16	16
**2** (rac^a^)	4	4
**2a** (−)-(*R,R*)	4	4
**2b** (+)-(*S,S*)	4	4
**3** (rac^a^)	2	2
**3a** (−)-(*R,R*)	4	4
**3b** (+)-(*S,S*)	4	4
**4** (rac^a^)	>128	>128
**4a** (−)-(*R,R*)	>128	>128
**4b** (+)-(*S,S*)	>128	>128
Vancomycin^b^	0.7	0.7

a Racemic mixture.

b Reference compound.

### Enantiomeric Separation of the Four Other Main Scaffolds Obtained by Biotransformation

After successfully applying this methodology to the four *trans*-δ-viniferin derivatives, we used it to separate the enantiomers of the other main scaffolds (Figure 1). To our knowledge, the enantiomeric separation of these four scaffolds has never been reported before. The subsequently obtained ECD spectra can be used as references for future research, including studies on plant metabolites since these families are present in nature (except acyclic dimers) (Khan et al., 1986; Lins et al., 1991; Ohyama et al., 1995; Pezet et al., 2003).

Since compounds **1**–**8** share common structural features, we decided to continue with the same stationary phase used in the study of the *trans*-δ-viniferins **1**–**4** to separate the enantiomers of compounds **5**–**8**. However, the efficiency of the column was not the same for these other scaffolds and the separation of these compounds at the milligram level was not possible. Fortunately, ECD measurements require concentrations of about 20 µM for these types of compounds, which represents a mass requirement of about 20 µg (molar mass of about 500 g/mol). These quantities are compatible with small volume injections to maintain the separation achieved between the chromatographic peaks. For each compound (**5**–**8**), repeated injections with manual collection were performed to obtain the pure enantiomers. Each enantiomer was monitored with UHPLC-PDA-ELSD-MS, NMR and chiral-HPLC-PDA.

**FIGURE 4:**
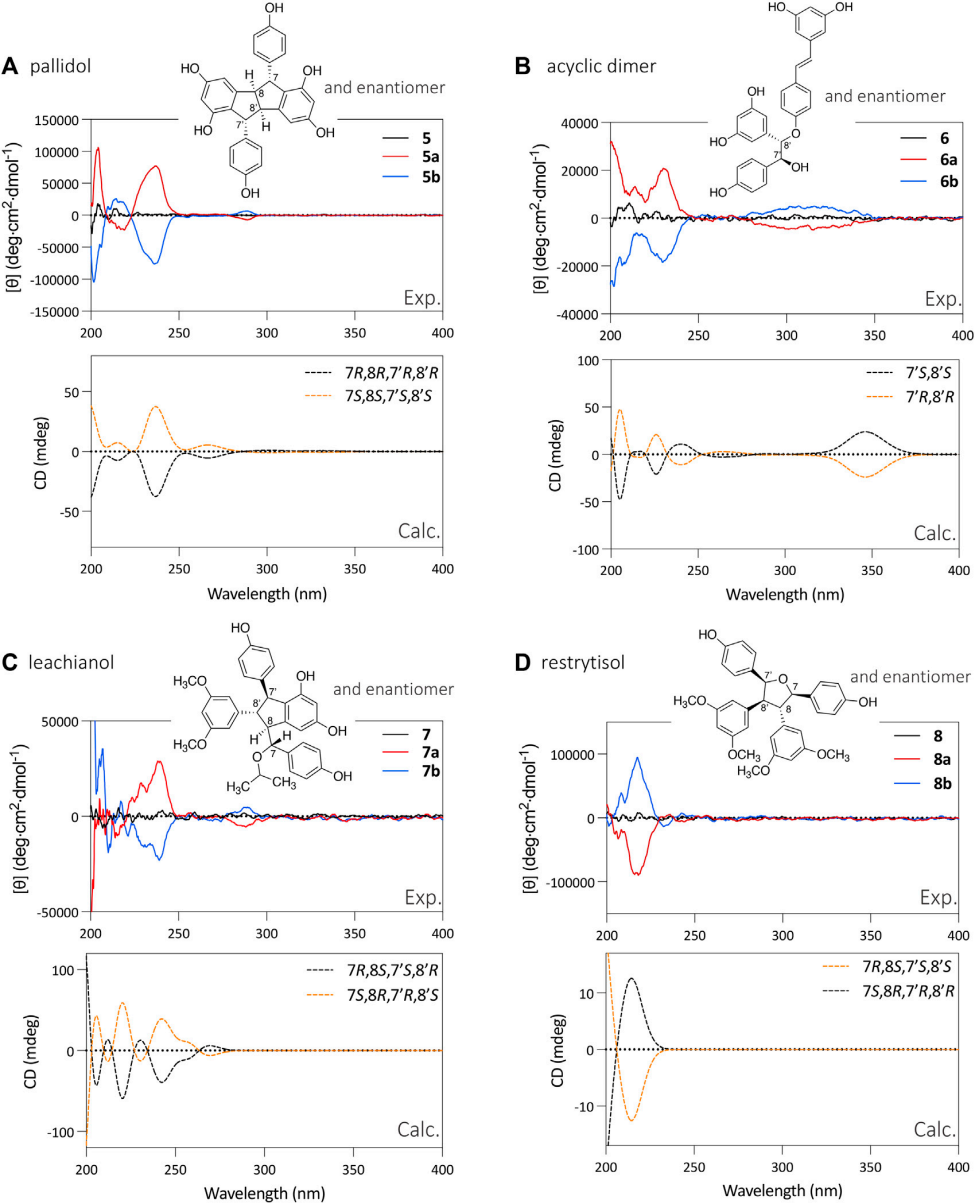
Relative configuration, experimental ECD spectra and calculated ECD spectra of the enantiomeric pairs of compounds **5**–**8**. Experimental red curve matches with the calculated orange one, while the experimental blue curve matches with the experimental black curve. **(A)** pallidol-type **5**. **(B)** acyclic dimer-type **6**. **(C)** leachianol-type **7**. **(D)** restrytisol-type **8**.

As explained above, the NMR analysis of compounds **5**, **6**, **7**, and **8** was used to determine their relative stereochemistry. The purified enantiomers **5a**, **5b**, **6a**, **6b**, **7a**, **7b, 8a,** and **8b** were subjected to ECD analysis to determine the absolute configuration of the chiral carbons. Analysis of the ECD spectra showed opposite curves with identical magnitude, indicating efficient chiral separation of the two optically pure enantiomers (Figure 4).

To determine the absolute configuration of the chiral carbons in each structure, the 3D structures of the most stable conformers of each enantiomer were calculated using TD-DFT at B3LYP/def2svp//B3LYP/6–31G (d,p) level and a SCRF model in MeCN. After this step, the ECD spectra of the two enantiomers were simulated and compared with the experimental ones (Figure 4).

For example, the calculated ECD spectrum for the (7*R*,8*R*,7′*R*,8′*R*) stereoisomer of compound **5** matches with the experimental data of **5a**, with negative CE around 200 and 235 nm, and a positive CE around 285 nm. Inherently to this type of calculations, some bands can be wrongly predicted, as it is the case for the experimental positive CE around 215 nm, predicted as negative. The other stereoisomer, the (7*S*,8*S*,7′*S*,8′*S*) displayed a mirror image. This comparison resulted in the attribution of the (7*R*,8*R*,7′*R*,8′*R*) configuration to **5a** and (7*S*,8*S*,7′*S*,8′*S*) configuration to **5b** (Figure 4).

Using the same approach, it was possible to assign the absolute configuration of **6a** as (7′*R*,8′*R*) and **6b** as (7*′S*,8′*S*)*.* The absolute configuration of **7a** was determined as (7*R*,8*S*,7′*S*,8′*R*) and **7b** as (7*S*,8*R*,7′*R*,8′*S*). Finally, the absolute configuration of **8a** was assigned as (7*R*,8*S*,7′*S*,8′*S*) and **8b** as (7*S*,8*R*,7′*R*,8′*R*) (Figure 4). A summary for each compound with the chromatographic and spectroscopic details can be found on Supplementary Figure S6 (compound **5**), Supplementary Figure S7 (compound **6**), Supplementary Figure S8 (compound **7**) and Supplementary Figure S9 (compound **8**).

## Conclusion

In conclusion, the use of a chiral column allowed the isolation and characterization of the enantiomeric pairs of each of the five main scaffolds obtained by the stilbene biotransformation process using the *B. cinerea* Pers. enzymatic secretome. This study confirms that the reactions probably mediated by laccases leading to these compounds are not enantioselective, resulting in racemic mixtures.

Interest was directed to the *trans*-δ-viniferin series (**1**–**4**), as some of these compounds have shown remarkable activities against methicillin-resistant strains of *S. aureus*. Chiral separation was used to isolate and test enantiomeric pairs of these compounds. The results showed that the enantiomers have similar biological activities as the racemic mixtures. This suggests that the uptake and the bacterial target of these compounds are not affected by the absolute configuration. On a practical point of view, this also indicates that further studies will not need to have pure enantiomers since the racemic mixture has the same activity. Ongoing studies on the mode of action of these compounds will attempt to elucidate this important issue.

## Experimental Section

### General Experiment Procedures

The specific rotations were measured in MeCN on a JASCO P-1030 polarimeter (Loveland, CO, United States) in a 10 cm tube. The UV and ECD spectra were recorded on a JASCO J-815 spectrometer (Loveland, CO, United States) in MeCN, using a 1 cm cell. The scan speed was set at 200 nm/min in continuous mode between 500 and 190 nm, with a band width of 1 nm, a data pitch of 0.1 nm and 2 accumulations. NMR spectroscopic data were recorded on a Bruker Avance Neo 600 MHz NMR spectrometer equipped with a QCI 5 mm cryoprobe and a SampleJet automated sample changer (Bruker BioSpin, Rheinstetten, Germany). ^1^H NMR experiments were recorded in DMSO-*d*_6_. Chemical shifts are reported in parts per million (δ) and coupling constants (*J*) in Hz. The residual DMSO-*d*_6_ signal (δ_H_ 2.50); was used as internal standard for ^1^H. Chromatographic data were obtained on an Ultra-high-performance liquid chromatography system equipped with a photodiode array, an evaporative light-scattering detector, and a single quadrupole detector using heated electrospray ionization (UHPLC-PDA-ELSD-MS) (Waters, Milford, MA, United States). The ESI parameters were the following: capillary voltage 800 V, cone voltage 15 V, source temperature 120°C, and probe temperature 600°C. Acquisition was done in negative ionization mode (NI) with an *m/z* range of 150–1000 Da. The chromatographic separation was performed on an Acquity UPLC BEH C_18_ column (50 × 2.1 mm i. d., 1.7 μm; Waters) at 0.6 ml/min, 40°C with H_2_O (A) and MeCN (B) both containing 0.1% formic acid as solvents. The gradient was carried out as follows: 5–100% B in 7 min, 1 min at 100% B, and a re-equilibration step at 5% B for 2 min. The ELSD temperature was fixed at 45°C, with a gain of 9. The PDA data were acquired from 190 to 500 nm, with a resolution of 1.2 nm. The sampling rate was set at 20 points/s.

### Compounds Generation

The compounds **1**–**8** used in this experiment were obtained by the biotransformation of resveratrol and pterostilbene by the enzymatic secretome of *Botrytis cinerea* Pers., as described in our previous article (Righi et al., 2020). Their purity was controlled by UHPLC-PDA-ELSD-MS and NMR.

### Enantiomers Separation by Chiral HPLC

Compounds **1**–**4** were screened on different Daicel chiral columns to find the most efficient stationary phase: IA, IB-N5, IC, ID, IE, IF, IG, and IH (https://chiraltech.com for the details about the stationary phases). A first screening was performed with heptane/alcohol mixtures (EtOH or isopropanol) with 0.1% diethylamine. A second screening was performed with heptane/ethyl acetate, heptane/dichloromethane and heptane/tetrahydrofuran mixtures with 0.1% diethylamine. A last screening was performed with acetonitrile or methanol/ethanol mixtures with 0.1% diethylamine. The selected stationary phase was a cellulose substituted with *tris* (3,5-dimethylphenylcarbamate) immobilized on a 5 μm silica-gel (Chiralpak^®^ IB N-5 250 × 4.6 mm i. d., 5vµm; Daicel, Osaka, Japan). Compounds **1**–**8** were then injected on a HP 1100 Agilent High-Performance liquid chromatography equipped with a photodiode array detector (HPLC-PDA) (Agilent technologies, Santa Clara, CA, United States). The chromatographic separations were performed on the previously selected column Chiralpak^®^ IB N-5 column (250 × 4.6 mm i. d., 5 μm; Daicel) at 1 ml/min, with heptane (A) and EtOH (B) both containing 0.1% diethylamine as solvents. Solutions at 5–15 mg/ml of compounds **1**–**8** in dichloromethane/ethyl acetate mixtures were first injected to determine the best isocratic condition for each compound. Increasing volumes were injected until reaching the limit of separation. Repeated injections were finally performed with manual collection of the compounds in glass vials after the PDA detector. Separated enantiomers were controlled by UHPLC-PDA-ELSD-MS, chiral-HPLC-PDA and NMR.

### Electronic Circular Dichroism Calculations

The absolute configuration of the compounds was assigned according to the comparison of the calculated and experimental ECDs. The structures and the relative configurations proposed by NMR were energetically optimized through 3LYP/6–31G (d, p) in Gaussian 16 (© 2015–2022, Gaussian inc.). All possible conformers were calculated in Avogadro software (Version 1.2.0, © 2018 Avogadro Chemistry) through a Random rotor search algorithm. All conformers were energetically optimized through 3LYP/6–31G (d) in Gaussian 16 using a SCRF model in MeCN. Then, the conformers with the lowest energy (cut-off of 4 kcal/mol) were selected and checked to avoid imaginary frequencies. The conformers were selected and submitted to Gaussian16 software for ECD calculations, using TD-DFT with B3LYP/def2svp as basis set with SCRF model in MeCN. The computation in Gaussian was performed at the University of Geneva on the Baobab cluster (https://plone.unige.ch/distic/pub/hpc/baobab_en, accessed on 19 November 2021). The calculated ECD spectrum was generated in SpecDis1.71 software (Version 1.71, T. Bruhn, Berlin, Germany, 2017) based in Boltzmann weighing average.

### Description of the Isolated Compounds

*Trans*-δ-viniferin (**1**): [α]^25^_D_ 0.8 (c 0.06, MeCN). UV (MeCN) λmax (log ε) 227 (sh) (4.46), 285 (4.08), 312 (4.32), 333 (4.127), 350 (sh) (3.99) nm. ^1^H NMR (DMSO-*d*_6_, 600 MHz) δ 4.45 (1H, d, *J* = 7.9 Hz, H-8′), 5.39 (1H, d, *J* = 7.9 Hz, H-7′), 6.04 (2H, d, *J* = 2.2 Hz, H-10′, H-14′), 6.10 (1H, t, *J* = 2.5 Hz, H-12′), 6.11 (1H, t, *J* = 2.2 Hz, H-12), 6.38 (2H, d, *J* = 2.1 Hz, H-10, H-14), 6.77 (2H, d, *J* = 8.6 Hz, H-3′, H-5′), 6.84 (1H, d, *J* = 16.3 Hz, H-8), 6.89 (1H, d, *J* = 8.3 Hz, H-5), 6.99 (1H, d, *J* = 16.3 Hz, H-7), 7.19 (2H, d, *J* = 8.6 Hz, H-2′, H-6′), 7.23 (1H, s, H-2), 7.42 (1H, dd, *J* = 8.2, 1.9 Hz, H-6), 9.18 (2H, s, 11OH, 13OH), 9.21 (2H, s, 11′OH, 13′OH), 9.53 (1H, s, 4′OH). HR-ESI/MS analysis: *m/z* 453.1348 [M-H]^-^, (calcd for C_28_H_21_O_6_, 453.1338, ∆ = 2.2 ppm). MS/MS spectrum: CCMSLIB00009918864.

(-)-(*R,R*)-*trans*-δ-viniferin (**1a**): heptane:EtOH 82:18 + 0.1% DEA, *t*_R_ 30 min [α]^25^_D_ −19.6 (c 0.1, MeCN). ECD (c 22 µM, MeCN) λmax ([θ]) 214 (+33168), 233 (−11271), 272 (−6268), 299 (−4705), 335 (+3558) nm. UV (MeCN) and ^1^H NMR (DMSO-*d*_6_, 600 MHz): identical to compound **1**.

(+)-(*S,S*)-*trans*-δ-viniferin (**1b**): heptane:EtOH 82:18 + 0.1% DEA, *t*_R_ 46 min [α]^25^_D_ +15.4 (c 0.1, MeCN). ECD (c 18 µM, MeCN) λmax ([θ]) 214 (−42728), 232 (+8421), 274 (+6444), 298 (+6205), 332 (−4617) nm. UV (MeCN) and ^1^H NMR (DMSO-*d*_6_, 600 MHz): identical to compound **1**.

11′,13′-di-*O*-methyl-*trans*-δ-viniferin (**2**): [α]^25^_D_ 0.5 (c 0.07, MeCN). UV (MeCN) λmax (log ε) 227 (sh) (4.45), 285 (4.06), 312 (4.30), 333 (4.25), 350 (sh) (3.97) nm. ^1^H NMR (DMSO-*d*_6_, 600 MHz) δ 3.70 (6H, s, CH_3_O-11′, CH_3_O-13′), 4.58 (1H, d, *J* = 8.1 Hz, H-8′), 5.56 (1H, d, *J* = 8.1 Hz, H-7′), 6.10 (1H, t, *J* = 2.3 Hz, H-12), 6.36 (4H, 2xd, *J* = 2.3 Hz, H-10, H-14, H-10′, H-14′), 6.43 (1H, t, *J* = 2.3 Hz, H-12′), 6.76 (2H, d, *J* = 8.5 Hz, H-3′, H-5′), 6.82 (1H, d, *J* = 16.3 Hz, H-8), 6.90 (1H, d, *J* = 8.4 Hz, H-5), 6.97 (1H, d, *J* = 16.3 Hz, H-7), 7.19 (3H, m, H-2, H-2′, H-6′), 7.42 (1H, dd, J = 8.4, 1.9 Hz, H-6), 9.16 (2H, s, 11OH, 13OH), 9.52 (1H, s, 4′OH). HR-ESI/MS analysis: *m/z* 481.1652 [M-H]^-^, (calcd for C_30_H_25_O_6_, 481.1651, ∆ = 0.2 ppm). MS/MS spectrum: CCMSLIB00009918871.

(-)-(*R,R*)-11′,13′-di-*O*-methyl-*trans*-δ-viniferin (**2a**): heptane:EtOH 80:20 + 0.1% DEA, *t*_R_ 23 min [α]^25^_D_ −19.7 (c 0.06, MeCN). ECD (c 17 µM, MeCN) λmax ([θ]) 216 (+31567), 231 (−20076), 272 (−4156), 298 (−2064), 326 (+8532) nm. UV (MeCN) and ^1^H NMR (DMSO-*d*_6_, 600 MHz): identical to compound **2**.

(+)-(*S,S*)-11′,13′-di-*O*-methyl-*trans*-δ-viniferin (**2b**): heptane:EtOH 80:20 + 0.1% DEA, *t*_R_ 42 min [α]^25^_D_ 20.8 (c 0.06, MeCN). ECD (c 17 µM, MeCN) λmax ([θ]) 215 (−39094), 231 (+19255), 270 (+6091), 300 (+4294), 324 (−7083) nm. UV (MeCN) and ^1^H NMR (DMSO-*d*_6_, 600 MHz): identical to compound **2**.

11,13-Di-*O*-methyl-*trans*-δ-viniferin (**3**): [α]^25^_D_ −0.8 (c 0.07, MeCN); UV (MeCN) λmax (log ε) 227 (sh) (4.48), 285 (4.10), 312 (4.34), 333 (4.30), 350 (sh) (4.00) nm. ^1^H NMR (DMSO-*d*_6_, 600 MHz) δ 3.75 (6H, s, CH_3_O-11, CH_3_O-13), 4.46 (1H, d, *J* = 8.3 Hz, H-8′), 5.41 (1H, d, *J* = 8.3 Hz, H-7′), 6.04 (2H, d, *J* = 2.2 Hz, H-10′, H-14′), 6.10 (1H, t, *J* = 2.2 Hz, H-12′), 6.35 (1H, t, *J* = 2.2 Hz, H-12), 6.73 (2H, d, J = 2.2 Hz, H-10, H-14), 6.76 (2H, d, *J* = 8.6 Hz, H-3′, H-5′), 6.90 (1H, d, *J* = 8.3 Hz, H-5), 6.96 (1H, d, *J* = 16.4 Hz, H-8), 7.19 (2H, d, *J* = 8.6 Hz, H-2′, H-6′), 7.22 (1H, d, *J* = 16.4 Hz, H-7), 7.23 (1H, d, *J* = 1.9 Hz, H-2), 7.44 (1H, dd, *J* = 8.3, 1.9 Hz, H-6), 9.22 (2H, s, 11′OH, 13′OH), 9.53 (1H, s, 4′OH). HR-ESI/MS analysis: *m/z* 481.1654 [M-H]^-^, (calcd for C_30_H_25_O_6_, 481.1651, ∆ = 0.6 ppm). MS/MS spectrum: CCMSLIB00009918873.

(-)-(*R,R*)-11,13-di-*O*-methyl-*trans*-δ-viniferin (**3a**): heptane:EtOH 80:20 + 0.1% DEA, *t*_R_ 14 min [α]^25^_D_ −15.4 (c 0.1, MeCN). ECD (c 20 µM, MeCN) λmax ([θ]) 215 (+23454), 233 (−15588), 275 (−6800), 297 (−4917), 327 (+8434) nm. UV (MeCN) and ^1^H NMR (DMSO-*d*_6_, 600 MHz): identical to compound **3**.

(+)-(*S,S*)-11,13-di-*O*-methyl-*trans*-δ-viniferin (**3b**): heptane:EtOH 80:20 + 0.1% DEA, *t*_R_ 38 min [α]^25^_D_ 13.8 (c 0.1, MeCN). ECD (c 18 µM, MeCN) λmax ([θ]) 214 (−27117), 233 (+10076), 274 (+5953), 298 (+4928), 333 (−7742) nm. UV (MeCN) and ^1^H NMR (DMSO-*d*_6_, 600 MHz): identical to compound **3**.

11,13,11′,13′-tetra-*O*-methyl-*trans*-δ-viniferin (**4**): [α]^25^_D_ -0.3 (c 0.1, MeCN); UV (MeCN) λmax (log ε) 227 (sh) (4.56), 285 (4.17), 311 (4.41), 333 (4.37), 350 (sh) (4.12) nm. ^1^H NMR (DMSO-*d*_6_, 600 MHz) δ 3.70 (6H, s, CH_3_O-11′OMe, CH_3_O-13′), 3.74 (6H, s, CH_3_O-11, CH_3_O-13), 4.61 (1H, d, *J* = 8.5 Hz, H-8′), 5.59 (1H, d, *J* = 8.5 Hz, H-7′), 6.35 (1H, t, *J* = 2.3 Hz, H-12), 6.38 (2H, d, *J* = 2.3 Hz, H-10′, H-14′), 6.43 (1H, t, J = 2.3 Hz, H-12′), 6.72 (2H, d, *J* = 2.3 Hz, H-10, H-14), 6.76 (2H, d, *J* = 8.6 Hz, H-3′, H-5′), 6.92 (1H, d, *J* = 8.3 Hz, H-5), 6.95 (1H, d, *J* = 16.4 Hz, H-8), 7.21 (4H, m, H-2, H-2′, H-6′, H-7), 7.45 (1H, dd, *J* = 8.3, 1.9 Hz, H-6), 9.52 (1H, s, 4′OH). HR-ESI/MS analysis: *m/z* 509.1968 [M-H]^-^, (calcd for C_32_H_29_O_6_, 509.1964, ∆ = 0.8 ppm). MS/MS spectrum: CCMSLIB00009918877.

(-)-(*R,R*)-11,13,11′,13′-tetra-*O*-methyl-*trans*-δ-viniferin (**4a**): heptane:EtOH 90:10 + 0.1% DEA, *t*_R_ 23 min [α]^25^_D_ −23.3 (c 0.1, MeCN). ECD (c 18 µM, MeCN) λmax ([θ]) 215 (+33824), 232 (−25600), 275 (−6522), 293 (−6686), 322 (+10724) nm. UV (MeCN) and ^1^H NMR (DMSO-*d*_6_, 600 MHz): identical to compound **4**.

(+)-(*S,S*)- 11, 13, 11′,13′-tetra-*O*-methyl-*trans*-δ-viniferin (**4b**): heptane:EtOH 90:10 + 0.1% DEA, *t*_R_ 36 min [α]^25^_D_ +19.9 (c 0.1, MeCN). ECD (c 18 µM, MeCN) λmax ([θ]) 217 (−31291), 235 (+18807), 274 (+4380), 292 (+5152), 323 (−8648) nm. UV (MeCN) and ^1^H NMR (DMSO-*d*_6_, 600 MHz): identical to compound **4**.

Pallidol (**5**): UV (MeCN) λmax (log ε) 228 (sh) (4.29), 283 (3.58) nm. ^1^H NMR (DMSO-*d*_6_, 600 MHz) δ 3.60 (2H, s, H-8, H-8′), 4.30 (2H, s, H-7, H-7′), 6.05 (2H, d, *J* = 2.0 Hz, H-12, H-12′), 6.38 (2H, d, *J* = 2.0 Hz, H-10, H-10′), 6.60 (4H, d, *J* = 8.7 Hz, H-2, H-2′, H-6, H-6′), 6.84 (4H, d, *J* = 8.7 Hz, H-3, H-3′, H-5, H-5′), 8.88 (2H, s, 13′OH, 13OH), 9.02 (2H, s, 11′OH, 11OH), 9.05 (2H, s, 4′OH, 4OH). HR-ESI/MS analysis: *m/z* 453.1346 [M-H]^-^, (calcd for C_28_H_21_O_6_, 453.1338, ∆ = 1.7 ppm). MS/MS spectrum: CCMSLIB00009918856.

(7*S*,8*S*,7′*S*,8′*S*)-pallidol (**5a**): heptane:EtOH 82:18 + 0.1% DEA, *t*_R_ 14 min. ECD (c 50 µM, MeCN) λmax ([θ]) 204 (+106533), 219 (−22928), 237 (77036), 289 (−7203) nm. UV (MeCN) and ^1^H NMR (DMSO-*d*_6_, 600 MHz): identical to compound **5**.

(7*R*,8*R*,7′*R*,8′*R*)-pallidol (**5b**): heptane:EtOH 82:18 + 0.1% DEA, *t*_R_ 18 min. ECD (c 50 µM, MeCN) λmax ([θ]) 202 (−105030), 214 (25694), 236 (−75882), 288 6358) nm. UV (MeCN) and ^1^H NMR (DMSO-*d*_6_, 600 MHz): identical to compound **5**.

*Threo*-resveratrol acyclic dimer (**6**): UV (MeCN) λmax (log ε) 228 (sh) (4.25), 285 (4.00), 306 (4.14), 323 (4.15), 340 (sh) (3.85) nm. ^1^H NMR (DMSO-*d*_6_, 600 MHz) δ 4.66 (1H, d, *J* = 6.5 Hz, H-7′), 5.00 (1H, d, *J* = 6.5 Hz, H-8′), 5.41 (1H, s, 7′OH), 5.97 (1H, t, *J* = 2.2 Hz, H-12′), 6.03 (2H, d, *J* = 2.2 Hz, H-10′, H-14′), 6.10 (1H, t, *J* = 2.2 Hz, H-12), 6.35 (2H, d, *J* = 2.2 Hz, H-10, H-14), 6.58 (2H, d, *J* = 8.6 Hz, H-3′, H-5′), 6.81 (1H, d, *J* = 16.2 Hz, H-8), 6.83 (2H, d, *J* = 8.8 Hz, H-3, H-5), 6.89 (1H, d, *J* = 16.2 Hz, H-7), 6.99 (2H, d, *J* = 8.6 Hz, H-2′, H-6′), 7.38 (2H, d, *J* = 8.8 Hz, H-2, H-6). HR-ESI/MS analysis: *m/z* 471.1451 [M-H]^-^, (calcd for C_28_H_23_O_7_, 471.1444, ∆ = 1.5 ppm). MS/MS spectrum: CCMSLIB00009918857.

(7′*R*,8′*R*)-*threo*-resveratrol acyclic dimer (**6a**): heptane:EtOH 70:30 + 0.1% DEA, *t*_R_ 13 min. ECD (c 39 µM, MeCN) λmax ([θ]) 201 (31309), 230 (20861), 315 (−4865) nm. UV (MeCN) and ^1^H NMR (DMSO-*d*_6_, 600 MHz): identical to compound **6**.

(7′*S*,8′*S*)-*threo*-resveratrol acyclic dimer (**6b**): heptane:EtOH 70:30 + 0.1% DEA, *t*_R_ 19 min. ECD (c 39 µM, MeCN) λmax ([θ]) 202 (−28697), 230 (−18357), 316 (5003) nm. UV (MeCN) and ^1^H NMR (DMSO-*d*_6_, 600 MHz): identical to compound **6**.

7-*O*-isopropyl-11′,13′-di-*O*-methylleachianol G (**7**): UV (MeCN) λmax (log ε) 228 (sh) (4.42), 283 (3.75), 323 (3.44) nm. ^1^H NMR (DMSO-*d*_6_, 600 MHz) δ 0.65 (3H, d, *J* = 6.1 Hz, CH_3_-7c), 0.85 (3H, d, *J* = 6.1 Hz, CH_3_-7b), 3.07 (1H, dd, *J* = 8.5, 2.4 Hz, H-8), 3.11 (1H, hept, J = 6.1 Hz, 7H-a), 3.43 (1H, t, *J* = 2.7 Hz, H-8′), 3.66 (6H, s, CH3O-11′, CH3O-13′), 4.02 (1H, d, *J* = 8.5 Hz, H-7), 5.31 (1H, d, *J* = 2.1 Hz, H-14), 6.08 (1H, d, *J* = 2.1 Hz, H-12), 6.11 (2H, d, *J* = 2.3 Hz, H-10′, H-14′), 6.30 (1H, t, *J* = 2.3 Hz, H-12′), 6.63 (2H, d, *J* = 8.5 Hz, H-3′, H-5′), 6.66 (2H, d, *J* = 8.4 Hz, H-3, H-5), 6.80 (2H, d, *J* = 8.5 Hz, H-2′, H-6′), 6.90 (2H, d, *J* = 8.4 Hz, H-2, H-6). HR-ESI/MS analysis: *m/z* 541.2222 [M-H]^−^, (calcd for C_33_H_33_O_7_, 541.2226, ∆ = 0.7 ppm). MS/MS spectrum: CCMSLIB00009918869.

(7*S*,8*R*,7′*R*,8′*S*)-*O*-isopropyl-11′,13′-di-*O*-methylleachianol G (**7a**): heptane:EtOH 83:17 + 0.1% DEA, *t*_R_ 8 min. UV (MeCN) λmax (log ε) 228 (sh) (4.49), 283 (3.78) nm. ECD (c 25 µM, MeCN) λmax ([θ]) 228 (17470), 238 (28892), 288 (−5405) nm. ^1^H NMR (DMSO-*d*_6_, 600 MHz): identical to compound 7.

(7*R*,8*S*,7′*S*,8′*R*)-*O*-isopropyl-11′,13′-di-*O*-methylleachianol G (**7b**): heptane:EtOH 83:17 + 0.1% DEA, *t*_R_ 9 min. UV (MeCN) λmax (log ε) 228 (sh) (4.49), 283 (3.78) nm. ECD (c 28 µM, MeCN) λmax ([θ]) 230 (−15943), 239 (−22974), 288 (4648) nm. ^1^H NMR (DMSO-*d*_6_, 600 MHz): identical to compound 7.

11.11′,13.13′-tetra-*O*-methylrestrytisol B (**8**): UV (MeCN) λmax (log ε) 228 (sh) (4.43), 283 (3.88), 323 (3.84) nm. ^1^H NMR (DMSO-*d*_6_, 600 MHz) δ 3.53 (6H, s, CH_3_O-11′OMe, CH_3_O-13′), 3.57 (1H, t, *J* = 10.5 Hz, H-8), 3.64 (6H, s, CH_3_O-11, CH_3_O-13), 4.12 (1H, overlapped, H-8′), 4.99 (1H, d, *J* = 10.0 Hz, H-7), 5.43 (1H, d, *J* = 9.0 Hz, H-7′), 6.04 (1H, t, *J* = 2.2 Hz, H-12′), 6.10 (2H, d, *J* = 2.2 Hz, H-10′, H-14′), 6.25 (1H, t, *J* = 2.2 Hz, H-12), 6.42 (2H, d, *J* = 2.2 Hz, H-10, H-14), 6.52 (2H, d, *J* = 8.5 Hz, H-3′, H-5′), 6.73 (2H, d, *J* = 8.5 Hz, H-3, H-5), 6.96 (2H, d, *J* = 8.5 Hz, H-2′, H-6′), 7.25 (2H, d, *J* = 8.5 Hz, H-2, H-6). HR-ESI/MS analysis: *m/z* 527.2075 [M-H]^-^, (calcd for C_32_H_31_O_7_, 527.2070, ∆ = 0.9 ppm). MS/MS spectrum: CCMSLIB00009918870.

(7*S*,8*R*,7′*R*,8′*R*)-11.11′,13.13′-tetra-*O*-methylrestrytisol B (**8a**): heptane:EtOH 81:19 + 0.1% DEA, *t*_R_ 14 min. UV (MeCN) λmax (log ε) 228 (sh) (4.43), 283 (3.64) nm. ECD (c 15 µM, MeCN) λmax ([θ]) 207 (−32706), 218 (−87507) nm. ^1^H NMR (DMSO-*d*_6_, 600 MHz): identical to compound **8**.

(7*R*,8*S*,7′*S*,8′*S*)- 11.11′,13.13′-tetra-*O*-methylrestrytisol B (8b): heptane:EtOH 81:19 + 0.1% DEA, *t*_R_ 18 min. UV (MeCN) λmax (log ε) 228 (sh) (4.43), 283 (3.64) nm. ECD (c 15 µM, MeCN) λmax ([θ]) 208 (37443), 217 (94041) nm. ^1^H NMR (DMSO-*d*_6_, 600 MHz): identical to compound **8**.

### Minimum Inhibitory Concentration Determinations

*Staphylococcus aureus* strains Newman (MSSA) and COL (MRSA) was grown in Muller-Hinton broth (MHB) for 7 h at 37°C with agitation. Microtiter plate wells containing two-fold dilutions of the test compounds in MHB medium were inoculated with approximately 10^5^ bacteria/well. After static incubation at 37°C for 16 h, MICs were determined as the first well without visible growth.

## Data Availability

The raw data files for the NMR, chiral-HPLC, UV and ECD (calculated and experimental) analysis are available at the following link: https://doi.org/10.26037/yareta:b7aq6opppjaddmzvb7vvqfdmca. The MS/MS spectrum of each isolated compound has its own accession number CCMSLIB00009918XXX on the Global Natural Product Social Molecular Networking (GNPS) (accessed via: https://gnps.ucsd.edu/ProteoSAFe/static/gnps-splash.jsp).
