# Supplementary material for: Chiral Separation of Stilbene Dimers Generated by Biotransformation for Absolute Configuration Determination and Antibacterial Evaluation
Source: Front Chem. 2022 May 31;10:912396. doi: 10.3389/fchem.2022.912396 (PMC9194554; doi:10.3389/fchem.2022.912396)
Supplement: Supplementary file 1 [file DataSheet1.pdf]

frontiers

Supplementary Material

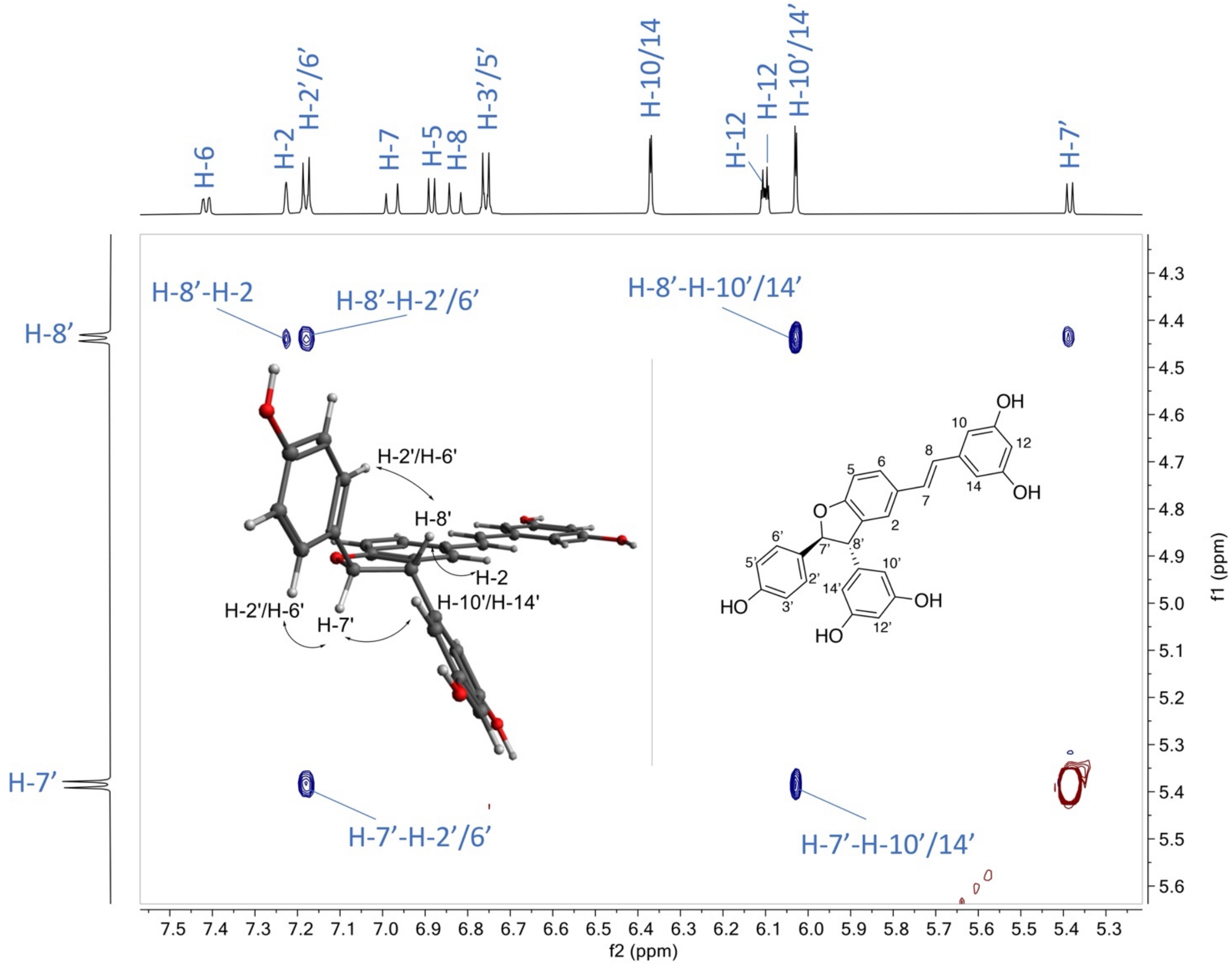

Supplementary Figure S1. ROESY NMR spectrum of *trans*-δ-viniferin **1** showing correlations allowing to assign the relative *trans* stereochemistry between H-7' and H-8'. The *trans*-δ-viniferin 3D structure with ROE correlations (left) and the structure with atom numbering (right).

trans-δ-viniferin (**1**)

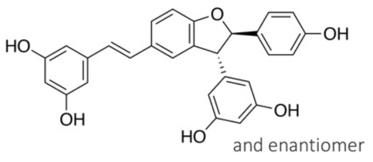

1

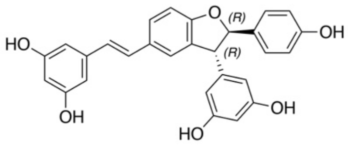

1a (-)-(*R*,*R*)

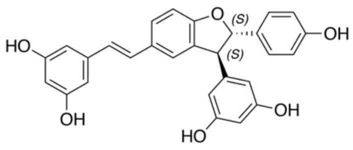

1b (+)-(*S*,*S*)

Chiralpak IB N-5 (250 x 4.6 mm i.d., 5 µm)
Mobile phase : heptane:EtOH 82:18
Detection : UV 310 nm

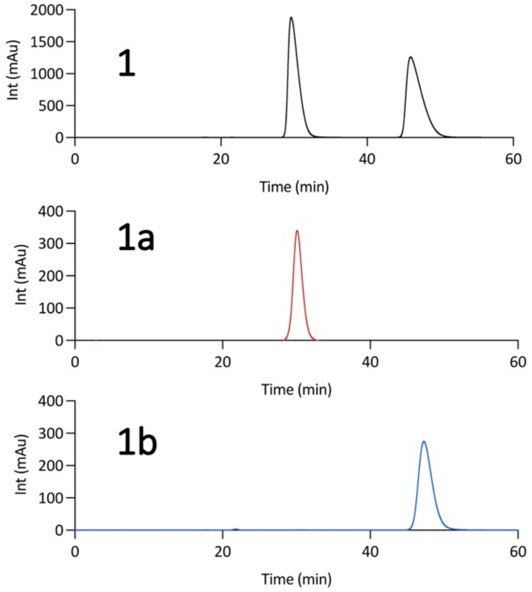

UV spectra

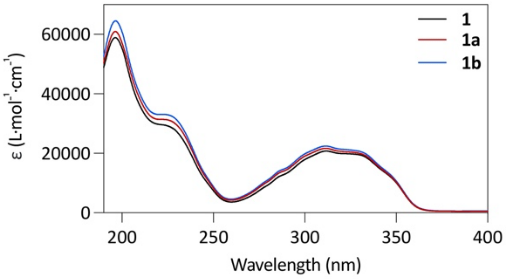

ECD spectra

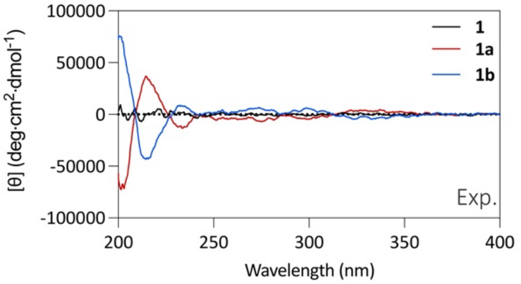

Optical rotation

	$[\alpha]^{25}_{D}$
**1**	+0.8
**1a**	-19.6
**1b**	+15.4

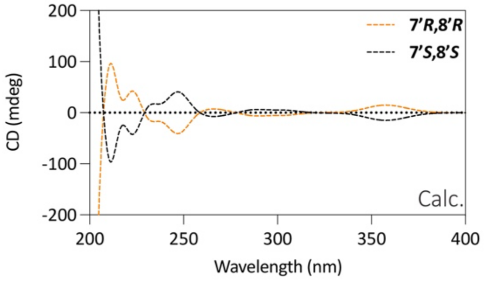

Supplementary Figure S2. Summary of the chromatographic (Chiral-HPLC) and spectroscopic (UV, ECD, specific rotation) details of compound **1** and its enantiomers **1a** and **1b**.

11',13'-Di-*O*-methyl-*trans*-δ-viniferin (**2**)

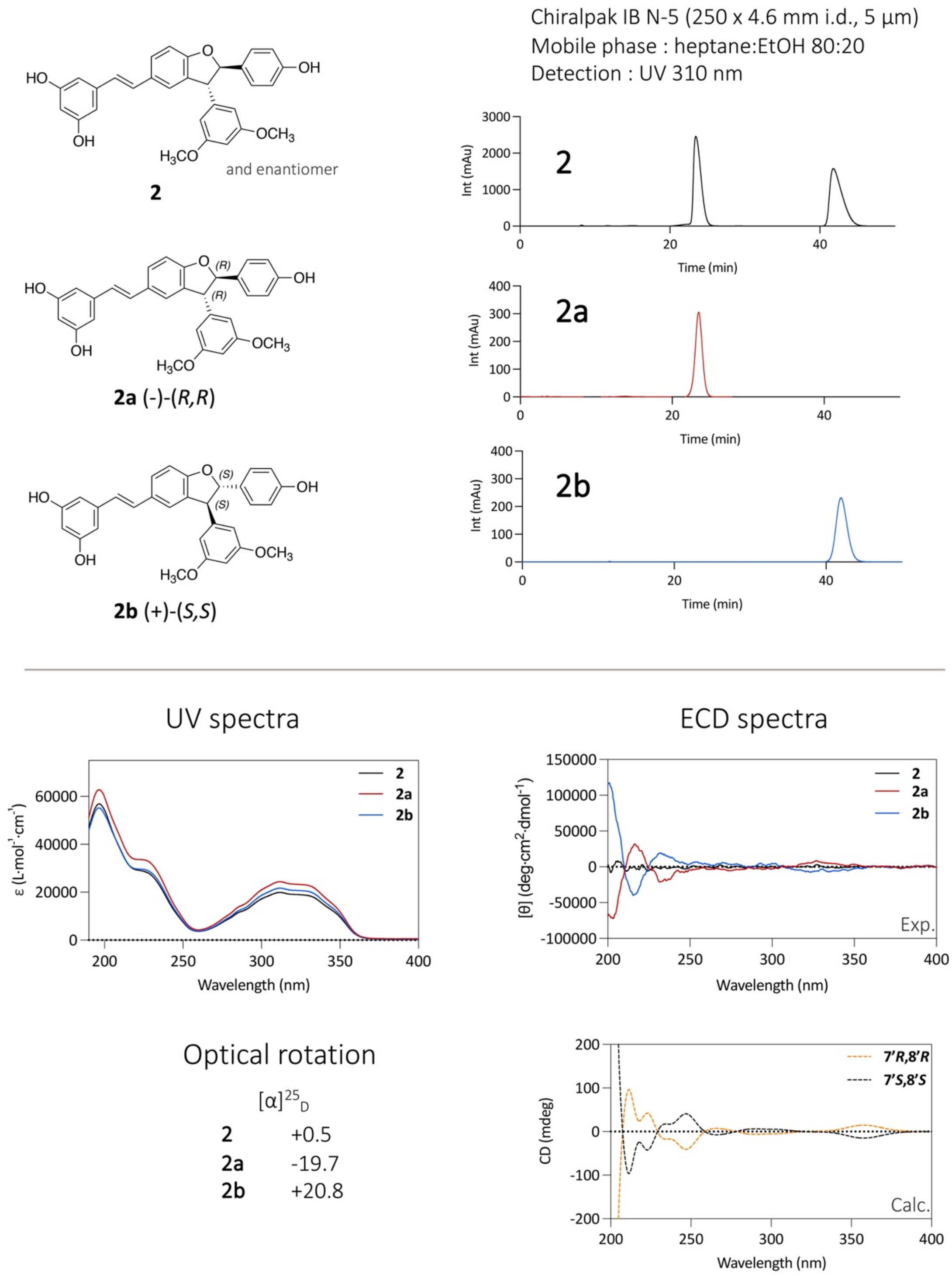

Supplementary Figure S3. Summary of the chromatographic (Chiral-HPLC) and spectroscopic (UV, ECD, specific rotation) details of compound **2** and its enantiomers **2a** and **2b**.

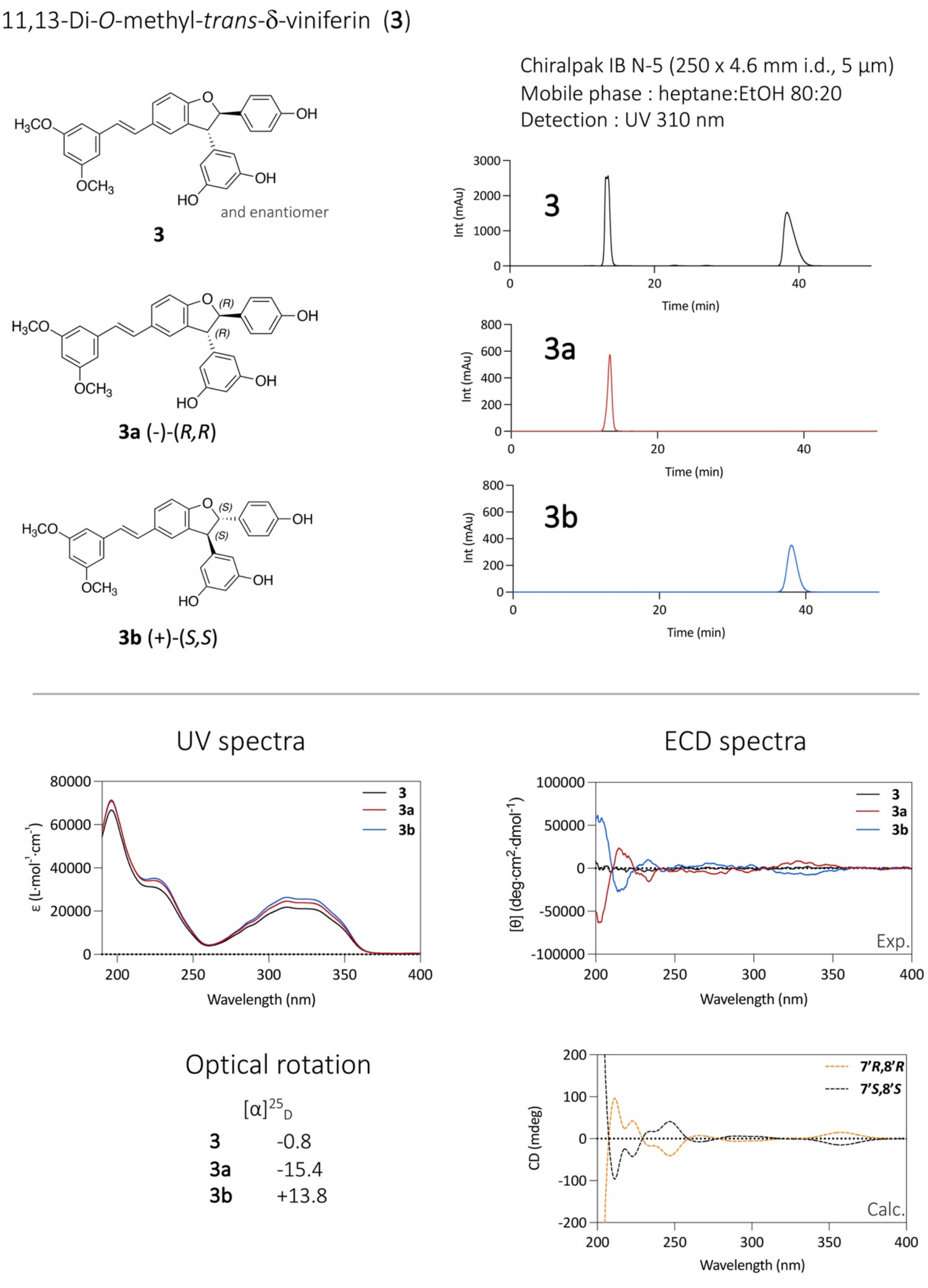

Supplementary Figure S4. Summary of the chromatographic (Chiral-HPLC) and spectroscopic (UV, ECD, specific rotation) details of compound **3** and its enantiomers **3a** and **3b**.

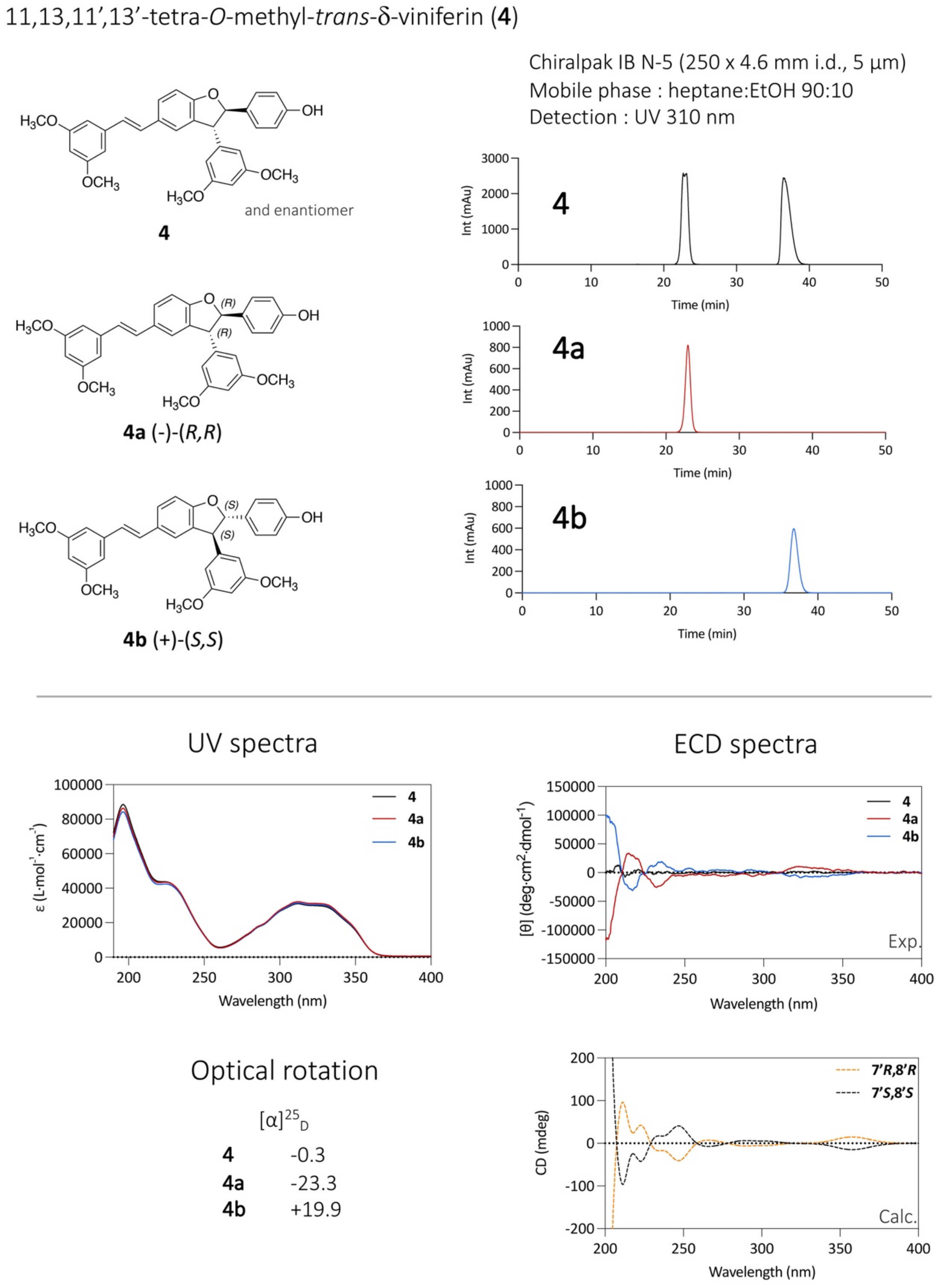

Supplementary Figure S5. Summary of the chromatographic (Chiral-HPLC) and spectroscopic (UV, ECD, specific rotation) details of compound **4** and its enantiomers **4a** and **4b**.

pallidol (**5**)

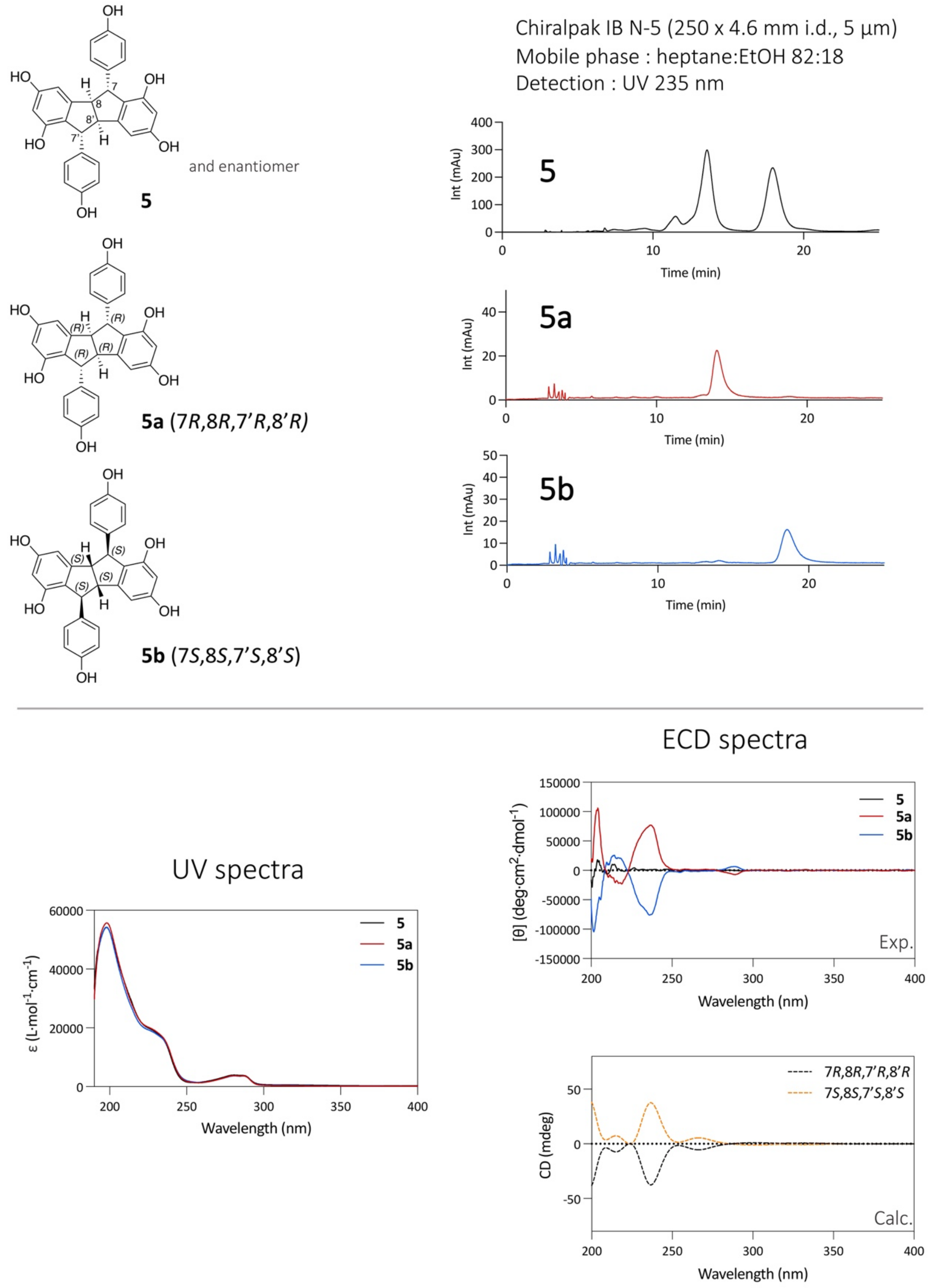

Supplementary Figure S6. Summary of the chromatographic (Chiral-HPLC) and spectroscopic (UV, ECD) details of compound **5** and its enantiomers **5a** and **5b**.

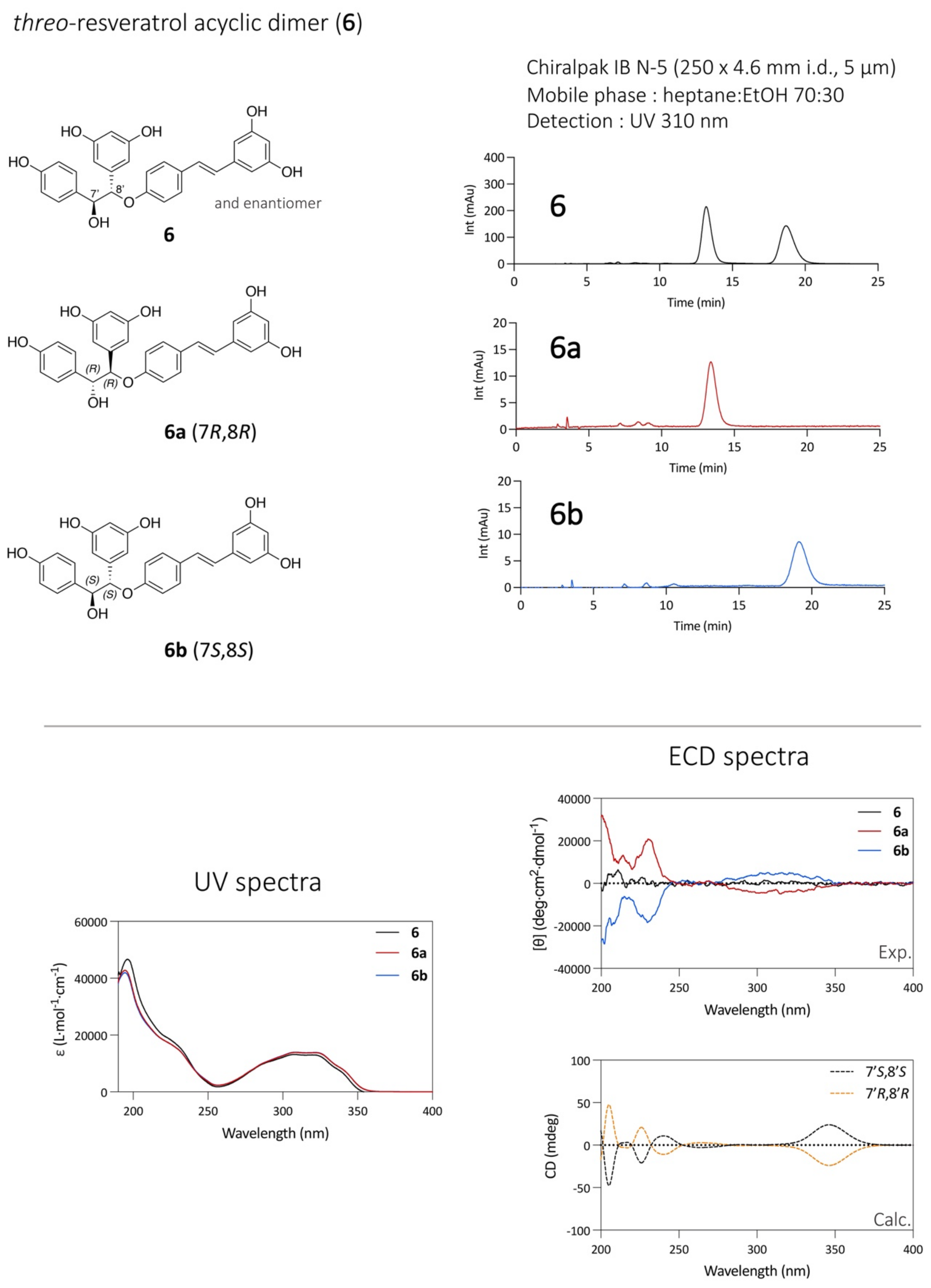

Supplementary Figure S7. Summary of the chromatographic (Chiral-HPLC) and spectroscopic (UV, ECD) details of compound **6** and its enantiomers **6a** and **6b**.

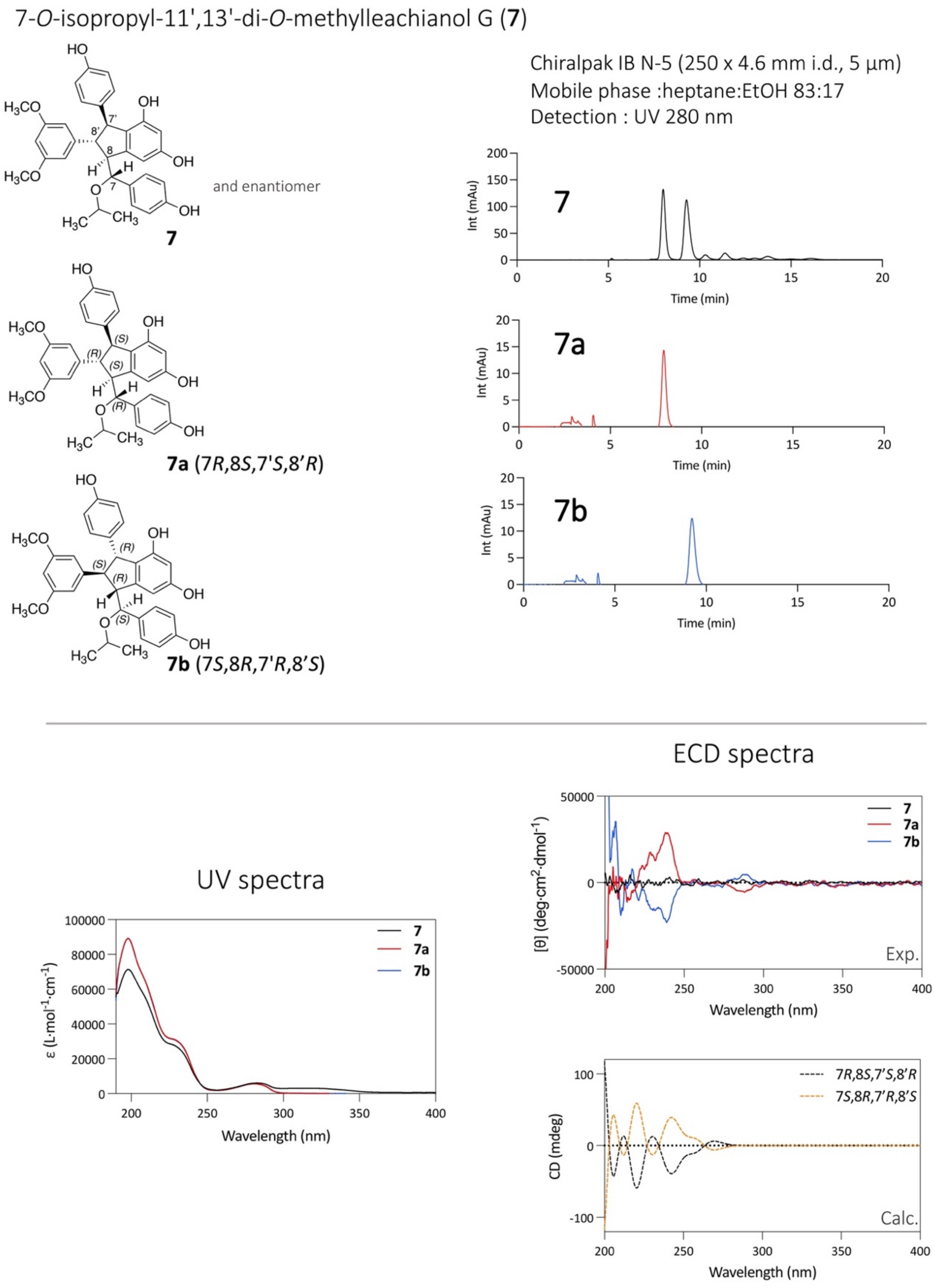

Supplementary Figure S8. Summary of the chromatographic (Chiral-HPLC) and spectroscopic (UV, ECD) details of compound **7** and its enantiomers **7a** and **7b**.

11,11',13,13'-tetra-*O*-methylrestrytisol B (**8**)

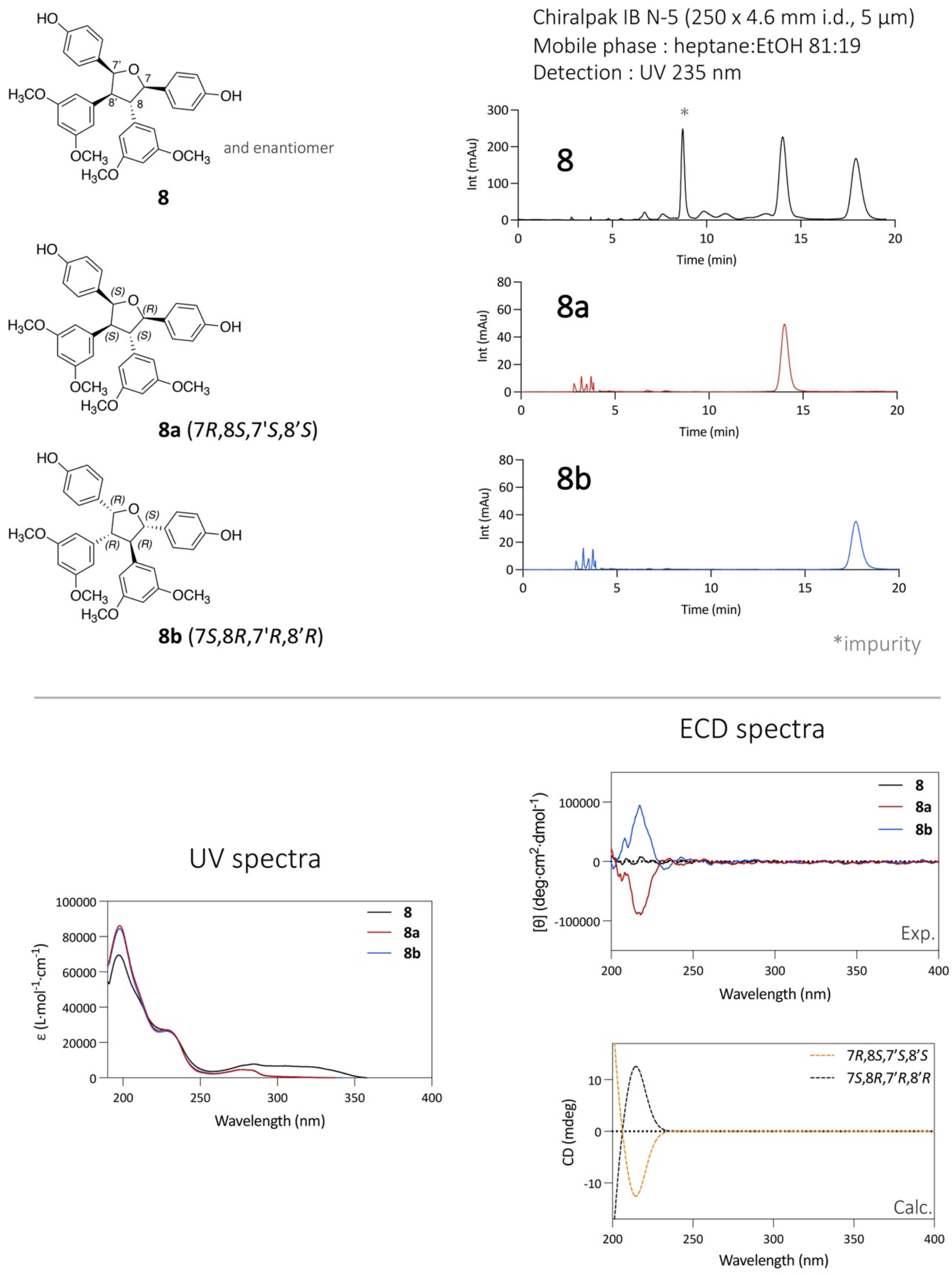

Supplementary Figure S9. Summary of the chromatographic (Chiral-HPLC) and spectroscopic (UV, ECD) details of compound **8** and its enantiomers **8a** and **8b**.
